# Urinary bisphenol A and pubertal development in Chinese school-aged girls: a cross-sectional study

**DOI:** 10.1186/s12940-017-0290-9

**Published:** 2017-07-27

**Authors:** Maohua Miao, Ziliang Wang, Xiaoqin Liu, Hong Liang, Zhijun Zhou, Hui Tan, Wei Yuan, De-Kun Li

**Affiliations:** 1 Key Laboratory of Reproduction Regulation of NPFPC, SIPPR, IRD, Fudan University, 779 Old Humin Road, Shanghai, 200237 China; 2 School of Public Health, Fudan University, Shanghai, 200032 China; 3 The National Centre for Register-based Research, Aarhus university, 8000 Aarhus, Denmark; 4 School of Public Health, Key Laboratory for Public Health Safety, Fudan University, Shanghai, 200032 China; 5 Department of Child and Adolescent Health, School of Public Health, Fudan University, Shanghai, 200032 China; 6 Division of Research, Kaiser Foundation Research Institute, Kaiser Permanente, Oakland, CA 94612 USA

**Keywords:** Bisphenol A, Endocrine disruptor, Girl, Puberty, Tanner stage

## Abstract

**Background:**

Animal studies suggest that bisphenol A (BPA) may perturb pubertal development in females. However, evidence from human studies is limited.

**Methods:**

This was a cross-sectional study to investigate the association between BPA exposure and pubertal development in school-aged girls. A total of 655 girls aged 9–18 years were selected from three schools in Shanghai, from May to June 2011. We collected one single spot urine sample from each girl. Urine BPA concentrations were measured by modified high-performance liquid chromatography and categorized according to LOD and the median of those above LOD. Pubertal development status was assessed by using Tanner staging, and age at menarche was collected as a milestone for mid-puberty. Modified Poisson regression was used to estimate adjusted prevalence ratios (PRs) and 95% confidence intervals (CIs).

**Results:**

After adjustment for potential confounders, girls with detected BPA were more likely to have delayed menarche, a mid-puberty event, compared with girls with undetectable BPA; the prevalence ratios (PR) were 0.73 (0.56, 0.95) for those with moderate BPA(LOD-median) and 0.72 (0.52, 0.99) for those with high BPA(>median), respectively. Girls aged 9–12 years with detected BPA were more likely to have reached pubic hair stage 2, the indicator of pubarche; while among girls aged >15 years, those with detected BPA were less likely to have reached pubic hair stage 5, the late stage of pubic hair development.

**Conclusions:**

BPA exposure was associated with alterations in the timing of pubertal development. Results in the present study should be interpreted with caution because of its cross-sectional nature and the limited sample size in each age group.

## Background

Puberty is a life stage with rapid physiological changes, including growth spurt and maturation of the gonads and the brain. Variations in pubertal timing and progression have been related to several antecedents, including genetic and environmental stressors [1]. Particularly, pubertal development has been reported to be accompanied with high vulnerability of the neuroendocrine system to environmental factors, which may lead to long-term effects on subsequent development [1]*.* One of the environmental stressors to developing children is bisphenol A (BPA) [2, 3]. BPA is a plasticizer, which is extensively used in resin-based and polycarbonate plastic products, including dental sealants and containers for foods and beverages. BPA can leach from a variety of consumer products and lead to wide spread human exposure [4].

Animal studies have shown that BPA acts as an endocrine disruptor with both estrogenic and anti-androgenic effects [2, 5]. The endocrine disrupting properties of BPA have also been demonstrated in human studies. These studies suggest that BPA is associated with increased risk of obesity [6], polycystic ovarian syndrome [7], recurrent miscarriage [8] and male infertility [2]. As pubertal growth is a hormone-dependent process [9], it is, therefore, plausible that chemicals with endocrine disrupting effects, like BPA, may induce altered pubertal maturation tempo. BPA is suggested to disrupt the key hormones responsible for sexual maturation [10]. Studies in mice have indicated that BPA exposure is associated with accelerated vaginal opening [3, 11, 12], but delayed vaginal opening [13] and testicular descent [14] have also been reported.

Pubertal development in humans involves both adrenarche and gonadarche, two independent processes. Adrenarche is induced by adrenal androgens, which are responsible for the initiation of pubic hair growth, as well as other pubertal events including acne, oily skin, deepened voice and adult-type body odor. Gonadarche refers to the reactivation of the hypothalamic-pituitary, and is characterized by the appearance of breast tissue. Longitudinal studies have shown that the pubertal development for girls may start with breast tissue appearance (thelarche) or pubic hair growth (pubarche), or concordant maturation of breasts and appearance of pubic hair [15, 16].

A few studies have explored the effect of BPA exposure on pubertal development in girls, but most of them used menarche age as the only endpoint for pubertal development assessment [17–19]*.* However, as mentioned above, breast development and the presence of pubic hair are known to be the early markers of pubertal maturation, while the onset of menarche occurs approximately 2 years after breast development. In addition, earlier pubertal onset may not necessarily be followed by an earlier age of menarche [20] due to the compensatory delay, which indicates both earlier and later events of pubertal development should be included to examine the effect of exogenous endocrine disrupting chemicals like BPA.

In the present study, we examined the profile of pubertal development in relation to BPA exposure among school-aged girls using menarche and Tanner stages, through which puberty onset and progression were evaluated.

## Methods

The present study was an ancillary study to a large national study of pubertal development and health of adolescents in schools in Jiading District, Shanghai, China, which had already collected anthropometric measures and information related to pubertal development. The current study added collection of urine samples. Therefore, participants were not aware of the specific hypothesis of the study. A detailed description of our study has been published elsewhere [6]. The following are brief descriptions of the study elements relevant to the present study.

### Study population

In 2011, the largest elementary, middle, and high schools in the study area (Jiading District, Shanghai) were selected, respectively. All girls from grades 4 through 12(aged from 9 to 18 years) were considered eligible for the study. We randomly selected four classes from each grade(i.e. elementary, middle, and high school) and about 80 girls from each grade were recruited (there were approximately 20 girls in each class). Overall, 743 girls were eligible for our study. Among them, ten girls (1.3%) refused to participate, 72 girls did not provide urine specimens, and urine samples of six girls were accidentally damaged during transportation. Finally, 655 girls (88.2%) were included in the analyses.

### In-person data collection

All girls completed a self-administered questionnaire, in which the following informations were collected:1) demographic characteristics, including age, school and residence. In addition, a 1–5 rating scale (poor to good) was used for self-evaluation of household income. Those who reported 1–3 were categorized as middle and below, and 4–5 were categorized as upper middle and highest. 2) Self-evaluated sleeping quality (poor, normal, good) and dietary patterns. A food frequency questionnaire was utilized to ascertain the frequency of food intake per week, including soy bean food (soy bean or soy bean products, such as soy bean milk, tofu, etc.), vegetables, fruits, junk food(food that have little nutritional value but plenty of calories, salt, and fats, like fried fast food and sugary carbonated beverages. The girls were asked to self-evaluate whether they have an unbalanced diet (yes/no). 3) Time spent on sports activity were collected and classified as ≥30 min/day and <30 min/day. 4) Girls’ current depression status was evaluated by using the published Children’s Depression Inventory (CDI).

### BPA measurements

We collected one single spot urine sample from each participating girl. We measured urinary concentrations of total BPA (free plus conjugated species) through modified high-performance liquid chromatography (HPLC) as described by He et al. [21]. The recommended value for the Limit of Detection (LOD) is three times the standard deviation of replicate measurements of a blank or low-level sample [22]. The LOD of BPA in the present study was 0.31 μg/L, which is comparable to the published LOD [4, 23]. Adjustment for creatinine was performed as urine BPA level divided by creatinine level to account for urine volume.

### Pubertal development assessment

Pubertal development was measured by Tanner staging, according to internationally accepted criteria, through visual inspection by the same trained physician [24]. Tanner staging provides separate scales for pubic hair and breast development. Pubic hair and breast development was determined as 1(pre-puberty), 2(onset), 3(ongoing), 4(nearly complete) or 5 (complete and adult-like) [25]. Height and weight were measured while barefoot and clad only in light underwear, according to the National Health and Nutrition Examination Survey (NHANES) [26].

### Statistical analysis

All analyses were conducted using STATA 12.0 (Stata Corp., LP, College Station, TX). Demographic characteristics of girls were tabulated according to their urine BPA level, which was categorized into three groups by LOD(0.31 μg/L) and the median of those above LOD(3.75 μg/gCr). This categorization can provide larger sample size when examining the effect of BPA at higher and lower exposure level compared with other categorization alternatives. We did not examine pubertal stages as ordinal categorical variables since the pubertal development did not follow a linear velocity, largely due to the growth spurt. Instead, we tabulated the percentage of girls who reached pubertal milestones across BPA exposure level at the relevant age. We examined associations between BPA exposure and different milestones (present or absent) representing the onset of pubertal development, as indicated by breast development stage 2 and above(B2+) and pubic hair stage 2 and above(PH2+), mid-puberty, as indicated by menarche, and late puberty, as indicated by breast development stage 5 (B5) and pubic hair stage 2 (PH5). Inclusion of Tanner Stage 5 in the analysis would provide information on BPA’s effect on pubertal progression. Modified Poisson regression with robust error variance was used to estimate adjusted prevalence ratios (PRs) and 95% confidence intervals (CIs), since the outcomes (pubertal stages) were not rare and thus odds ratios were not likely compatible with risk estimate.

We did not provide PR for each age due to the small sample size; instead, girls were grouped by age according to their relevance to the outcome examined. For example, when we examined BPA’s effect on pubertal onset, we only included girls younger than 12 years, since almost all girls beyond 12 years reached Tanner stage 2 and thus no valid comparison can be performed. While in the analysis of menarche, we restricted our analyses in girls who were aged less than 14 years of age, since all participants older than 14 reported to have experienced menarche, and the longer since menarche, the less indicative of the current exposure to BPA for the exposure at menarche.

The following characteristic were adjusted in the Poisson regression models as potential confounders according to previous literature [19, 27–29]: age, BMI(≥25, 18.5–24.9, or <18.5), household income (middle and below, or upper middle and highest), sleep quality (good, normal, or poor), unbalanced diet (yes or no), sports activity (≥30 min/day, or < 30 min/day), and depression scores (categorized by median: ≥10, or <10).

## Results

The mean age of 655 participants was 12.9 ± 2.7 years, which was younger than the 88 girls who were not included in the analysis (15.8 ± 2.4 years).

The median urinary BPA concentration was 1.24 (interquartile range: LOD-4.80) μg/gCr in girls. About 60% of the urinary samples had concentrations of BPA above LOD (0.31 μg/L). The median BPA concentration level among those with detected BPA was 3.75 μg/gCr, which was used as the cut-point of higher and lower BPA exposure.

Table 1 shows the characteristics of participating girls according to urine BPA level. Urine BPA levels were comparable with regard to school categories, residence, household income, BMI, sleep quality, sports activity, dietary pattern (unbalanced diet; junk food intake, vegetable intake, fruit intake, soybean food intake), and depression status. Girls aged 11–12 and 15–16 had higher urinary BPA, but no clear pattern was observed with regard to the distribution of BPA across age.

**Table 1:** Characteristics of Participating Girls According to BPA Level

Characteristic	N	BPA level(%)		
		<LOD	LOD-Median^a^	>Median
All	655	39.7	30.2	30.1
Age (years)				
9–10	162	43.8	29.0	27.2
11–12	160	36.3	28.1	35.6
13–14	124	37.9	39.5	22.6
15–16	137	29.9	33.6	36.5
17–18	72	59.7	15.3	25.0
School				
Elementary	171	43.3	28.1	28.7
Middle school	286	37.1	33.6	29.4
High school	198	40.4	27.3	32.3
Residence				
Rural	99	35.4	33.3	31.3
Urban	490	40.4	29.4	30.2
Missing	66	40.9	31.8	27.3
Household income				
Middle and below	479	39.0	29.7	31.3
Upper middle and highest	174	41.4	31.6	27.0
Missing	2	50.0	50.0	0.0
BMI				
< 18.5	286	42.3	31.1	26.6
18.5–25	330	36.7	29.7	33.6
≥ 25	39	46.2	28.2	25.6
Sleep quality				
Bad	35	45.7	25.7	28.6
Normal	194	40.7	31.4	27.8
Good	392	38.5	30.6	30.9
Missing	34	41.2	23.5	35.3
Sports activity				
< 30 min/day	426	39.4	31.7	28.9
≥30 min/day	217	41.0	26.3	32.7
Missing	12	25.0	50.0	25.0
Unbalanced diet				
No	349	41.3	30.1	28.6
Yes	266	38.7	30.8	30.5
Missing	40	32.5	27.5	40.0
Depression scores				
< 10	272	36.8	29.4	33.8
≥ 10	305	40.3	33.1	26.6
Missing	78	47.4	21.8	30.8
Junk food intake				
< 5 days/week	484	39.2	30.4	30.4
≥5 days/week	165	40.6	29.1	30.3
Missing	6	50.0	50.0	0.0
Vegetable intake				
Not everyday	187	34.8	32.6	32.6
Everyday	464	41.4	29.3	29.3
Missing	4	75.0	25.0	0.0
Fruit intake				
Not everyday	281	39.2	32.0	28.8
Everyday	372	40.3	29.0	30.7
Missing	2	0.0	0.0	100.0
Soy bean food intake				
Not everyday	483	41.0	29.6	29.4
Everyday	172	36.0	32.0	32.0

^a^The median BPA concentration level among those with detected BPA was 3.75 μg/gCr

All girls reported to have experienced menarche at age 14 (Table 2). The following analyses on BPA and menarche were thus conducted among girls aged <14(*n* = 383), since the comparison of menarche occurrence across BPA exposure is not feasible among girls aged ≥14. All girls reached thelarche(breast development stage 2 and above, B2+) at age 12. With regard to pubic hair development, the percentage of girls who had reached pubarche (pubic hair stage 2 and above, PH2+) was 92.5% for girls at age 12. At age 14, all girls reached PH2+. We therefore chose girls aged <12 for the analysis of BPA and puberty onset, since most of the girls aged ≥12 had reached puberty onset (100% for thelarche and 92.5% for pubarche). For the same reason, analyses on puberty progression(breast development stage 2, B5; pubic hair stage 5, PH5) were conducted among girls aged ≥13.

**Table 2:** Percentage of Girls in the Examined Pubertal Stage, by Age

Age	Menstrual	B2+	B5	PH2+	PH5
	n(%)	n(%)	n(%)	n(%)	n(%)
9	3(3.9)	60(76.0)	0(0.0)	4(5.0)	0(0.0)
10	2(2.5)	75(90.4)	0(0.0)	10(12.2)	0(0.0)
11	25(31.3)	78(97.5)	3(3.8)	35(43.8)	0(0.0)
12	51(64.6)	80(100.0)	12(15.0)	74(92.5)	0(0.0)
13	51(78.5)	65(100.0)	19(29.2)	63(96.9)	7(10.8)
14	59(100.0)	59(100.0)	18(30.5)	59(100.0)	6(10.2)
15	63(100.0)	63(100.0)	26(41.3)	63(100.0)	20(31.8)
16	74(100.0)	73(100.0)	32(43.8)	73(100.0)	22(30.1)
17	63(100.0)	63(100.0)	43(68.3)	63(100.0)	27(42.9)
18	9(100.0)	9(100.0)	7(77.8)	9(100.0)	5(55.6)

A total of 383 girls who were less than 14 years old were analyzed with regard to the association between BPA exposure and menarche. Compared with girls with undetected BPA, girls with moderate (LOD-median) and high (>median) BPA were less likely to have experienced menarche, PRs(95%CI) were 0.73 (0.56, 0.95) and 0.72 (0.52, 0.99), respectively, indicating that BPA exposure was associated with delayed age at menarche(Table 3). Analyses by age also show that girls with moderate and high BPA had lower numbers who had reached menarche at ages 11, 12 and 13 years (data not shown).

**Table 3:** Urine BPA Level in Relation to Menarche

Age(years)	BPA level	N	%	Adjusted PR^a^
9–13				
	<LOD	153	37.3	1(ref)
	LOD-median	114	32.5	0.73 (0.56, 0.95)
	>median	116	32.8	0.72 (0.52, 0.99)

^a^Adjusted for age, BMI, household income, sleep quality, sports activity, unbalanced diet, and depression score

Among girls aged less than 12 years, those with moderate BPA were more likely to have reached PH2+ after adjustment for potential confounders (PR: 1.48; 95%CI: 0.76, 2.88). Analyses by age also showed that those with moderate BPA consistently had higher percentages of those who experienced PH2+ at the age of 9, 10 and 11 years(data not shown). However, among girls aged more than 13 years, those with moderate and high BPA were less likely to have reached PH5, PRs(95%CI) were 0.73(0.44, 1.21) and 0.76 (0.46, 1.25), respectively (Table 4). Analyses by age also showed a similar pattern at the age of 13, 14, 15, and 16 years (data not shown). A similar, but less typical association, was observed for breast development (Table 5).

**Table 4:** Urine BPA Level in Relation to Pubic Hair Development

Age(years)	BPA level	N	%	Adjusted PR^a^
Pubic hair Stage 2 or higher				
9–11				
	<LOD	107	14.0	1(ref)
	LOD-median	69	23.2	1.48 (0.76, 2.88)
	>median	65	27.7	1.17 (0.60, 2.25)
Pubic hair Stage 5				
13–18				
	<LOD	130	33.9	1(ref)
	LOD-median	106	18.9	0.73 (0.44, 1.21)
	>median	96	24.0	0.76 (0.46, 1.25)

^a^Adjusted for age, BMI, household income, sleep quality, sports activity, unbalanced diet, and depression score

**Table 5:** Urine BPA Level in Relation to Breast Development

Age(years)	BPA level	N	%	Adjusted PR^a^
Breast Stage 2 or higher				
9–11				
	<LOD	107	85.1	1(ref)
	LOD-median	69	94.2	0.99 (0.89, 1.10)
	>median	66	86.4	0.91 (0.80, 1.04)
Breast Stage 5				
13–18				
	<LOD	130	51.5	1(ref)
	LOD-median	106	34.0	0.85 (0.62 1.15)
	>median	96	43.8	0.85 (0.63, 1.15)

^a^Adjusted for age, BMI, household income, sleep quality, sports activity, unbalanced diet, and depression score

## Discussion

In this cross-sectional study of school-aged girls, we found that BPA exposure was associated with delayed menarche, an indicator of mid-puberty. We also found a non-significant tendency that BPA exposure was associated with earlier pubic hair onset, and delayed progression of pubic hair development.

The association between BPA exposure and delayed menarche found in the present study was consistent with a previous study based on NHANES data, which reported moderate BPA exposure was associated with delayed menarche, although the association was non-significant [19]. Timing of menarche is influenced by many factors, including genetic background, ethnicity, obesity, nutrition and physical activity. In the last few decades, more and more studies have recognized EDCs, including dioxins, phthalates, organohalogens and PCBs, as important contributors influencing the timing of menarche, although the findings have not always been consistent. In the present study, we did not observe a stronger association among girls with high BPA exposure compared with those with moderate exposure, which is consistent with the report of the nonmonotonic dose–response effect of EDCs, including BPA [30]. The onset of menarche occurs approximately 2 years after breast development. In studies carried out in Spain, the United States, and Greece, early maturing girls were found to present a compensatory delay in pubertal progression [31–33]. These observations were consistent with our finding that those with high BPA exposure were more likely to reach pubic hair onset earlier while having a later menarche age and pubic hair stage 5.

The association of BPA exposure with early pubic hair onset, although not statistically significant, is consistent with the effect observed on other estrogen-like endocrine disruptors such as phthalates [1]. It was reported that pubertal hair development may rely on adrenal androgens, chiefly dehydroepiandrosterone (DHEA) [34], while breast development is more likely to be attributed to the effect of estrogen [35]. Higher BPA has been found to be associated with increased DHEA levels in both pre-adolescent girls and adult females [36, 37], which provides a potential underlying mechanism for the present finding. The result is also in line with the finding that BPA exposure is associated with polycystic ovarian syndrome [7], an abnormality characterized by the presence of hyper androgenism [38].

Although both breast development and presence of pubic hair are known to be early markers of pubertal maturation, breast development is more likely to be confounded by excess adiposity [39], which may also contribute to the non-significant association between BPA exposure and breast development.

It has been reported that prenatal growth is sensitive to extrogenous EDCs exposure. Our study suggests that pubertal development may also be sensitive to adverse effects of BPA [2]. Perinatal BPA exposure is associated with fetal growth as well as the growth pattern after birth, including pubertal growth. The present study is limited by the fact that perinatal exposure was not collected and thus its contribution to pubertal development, as well as its interaction with peripubertal exposure, cannot be evaluated. Longitudinal studies examining both perinatal and peripubertal BPA exposure are needed to clarify the effect.

The study has several strengths. First, we utilized both menarche and Tanner Staging, a well-validated tool for the assessment of pubertal stage, which provides more information than most studies with menarche as the only endpoint. Second, we were able to control for many potential confounders including depression status, sports activity strength, and dietary pattern.

Our study also has limitations. First, the cross-sectional design limited our ability to examine a causal relationship due to difficulties with temporality. Second, the sample size in each age group was small and the power to detect a difference, if it existed, was limited. Around 12% of girls were not included in the analysis due to refusal or loss of urine samples. As the study was an ancillary study to a national survey, eligible girls were unaware of the specific hypothesis of the study, thus selection bias is less likely to be a problem. Third, we collected only one single spot urine to reflect the BPA exposure. BPA is metabolized relatively rapidly, [40]. One single urine BPA measurement may not reflect average BPA exposures. Although girls may be exposed to BPA via oral ingestion, inhalation and dermal absorption, the predominant source of exposure was likely through diet. Since dietary patterns were relatively steady, the BPA level obtained in the present study was likely representative of the average BPA exposure level. Fourth, recall error or bias may exist for reporting retrospective events such as age at menarche. Since the participants were not aware of the specific hypothesis of the study, this recall error was likely to be non-differential misclassification, thereby potentially diluting the associations [41]. Lastly, the study was also limited by the low proportion of girls with detectable BPA(around 60%), although the rate was similar to two other Chinese studies with similar LOD (0.31μg/L) [21, 42].

## Conclusions

We observed that BPA exposure was associated with earlier pubertal onset and delayed pubertal progression, although prospective studies are needed to warrant the association.

## Abbreviations

BMI: Body mass index
BPA: Bisphenol A
CIs: Confidence intervals
LOD: LIMIT of detection
PRs: Prevalence ratios
